# Membrane lipid renovation in *Pseudomonas aeruginosa* ‐ implications for phage therapy?

**DOI:** 10.1111/1462-2920.16136

**Published:** 2022-08-14

**Authors:** Rhiannon Lyon, Rebekah A. Jones, Holly Shropshire, Isabel Aberdeen, David J. Scanlan, Andrew Millard, Yin Chen

**Affiliations:** ^1^ BBSRC Midlands Integrative Biosciences Training Partnership University of Warwick Coventry UK; ^2^ School of Life Sciences University of Warwick Coventry UK; ^3^ MRC Doctoral Training Partnership University of Warwick Coventry UK; ^4^ Department of Genetics and Genome Biology University of Leicester UK

## Abstract

*Pseudomonas aeruginosa* is an important Gram‐negative pathogen with intrinsic resistance to many clinically used antibiotics. It is particularly troublesome in nosocomial infections, immunocompromised patients, and individuals with cystic fibrosis. Antimicrobial resistance (AMR) is a huge threat to global health, with a predicted 10 million people dying from resistant infections by 2050. A promising therapy for combatting AMR infections is phage therapy. However, more research is required to investigate mechanisms that may influence the efficacy of phage therapy. An important overlooked aspect is the impact of membrane lipid remodelling on phage binding ability. *P. aeruginosa* undergoes changes in membrane lipids when it encounters phosphorus stress, an environmental perturbation that is likely to occur during infection. Lipid changes include the substitution of glycerophospholipids with surrogate glycolipids and the over‐production of ornithine‐containing aminolipids. Given that membrane lipids are known to influence the structure and function of membrane proteins, we propose that changes in the composition of membrane lipids during infection may alter phage binding and subsequent phage infection dynamics. Consideration of such effects needs to be urgently prioritised in order to develop the most effective phage therapy strategies for *P. aeruginosa* infections.

**List of Abbreviations**
AMR: antimicrobial resistance
CAMP: cationic antimicrobial peptide
CF: cystic fibrosis
CL: cardiolipin
DAG: diacyl glycerol
GP: glycerophospholipid
Kdo: 3‐deoxy‐d‐*manno*‐oct‐2‐ulosonic acid
LPS: lipopolysaccharide
OL: ornithine lipid
PA: phosphatidic acid
PC: phosphatidylcholine
PE: phosphatidylethanolamine
PG: phosphatidylglycerol
PI: phosphatidylinositol phosphate
PlcP: phospholipase C
TLR4: toll‐like receptor 4

## THE IMPORTANCE OF *PSEUDOMONAS AERUGINOSA* AS A PATHOGEN

*Pseudomonas aeruginosa* is a Gram‐negative opportunistic pathogen. It is a major pathogen in hospitals, being found on various surfaces and in water supplies with the potential to infect immunocompromised and vulnerable patients (Kizny Gordon *et al*., 2017; Gellatly and Hancock, 2013). Common infection sites for *P. aeruginosa* include burns and wounds, the urinary tract, bloodstream and the lungs (Morin *et al*., 2021). *P. aeruginosa* is especially associated with lung infections in those patients with cystic fibrosis (CF) and chronic obstructive pulmonary disease (Welp and Bomberger, 2020). In Europe, around 41% of adults with CF have chronic *P. aeruginosa* infection (Orenti *et al*., 2022) which is associated with increased morbidity and mortality (Jurado‐Martín *et al*., 2021).

CF is a genetic disease caused by mutations in a chloride ion channel present in the membranes of cells of the lungs, gut and pancreas (Scoffone *et al*., 2017). This causes abnormally thick mucus, which is difficult to clear from the lungs. As a result, pathogens that get into the lung are not cleared, leading to infection. Respiratory disease is the main cause of death in people with cystic fibrosis (Martin *et al*., 2016). Lung infections in CF patients caused by *P. aeruginosa* begin as recurrent, intermittent infections, but, as time goes on, they progress to become chronic infections (Folkesson *et al*., 2012; Maldonado *et al*., 2016). There is evidence that an undetected reservoir of *P. aeruginosa* exists in the nasal sinuses of supposedly recovered patients, and it is from this reservoir that their lungs become re‐infected (Hansen *et al*., 2012). The chronic inflammatory response to persistent *P. aeruginosa* infection leads to serious damage to lung tissue (Folkesson *et al*., 2012). As a result, individuals with CF have a much lower life expectancy than the general population, with babies born in the UK in 2020 expected to live to a median age of 47 (Keogh *et al*., 2019). This is comparable to a European study which showed that the median survival age for patients in the European Cystic Fibrosis Patients cohort is 51.7 (McKone et al., 2021).

*P. aeruginosa* infections of CF lungs persist despite high levels of antimicrobial therapy given to infected individuals. One of the contributing factors for this is that *P. aeruginosa* possesses several intrinsic antimicrobial resistance (AMR) mechanisms (Jurado‐Martín *et al*., 2021; Moradali *et al*., 2017), along with other adaptations to the CF lung environment detailed later in this review. *P. aeruginosa* possesses a Gram‐negative outer membrane that is highly impermeable, restricting the entry of antibiotics into the cell. If antibiotics do penetrate this barrier, it possesses genome‐encoded efflux pumps that can expel antibiotics (Shigemura *et al*., 2015; Dreier and Ruggerone, 2015). Further mechanisms of AMR can be acquired by horizontal gene transfer from other bacterial species or spontaneous mutation (Breidenstein *et al*., 2011) with examples including β‐lactamases (Llanes *et al*., 2013; Pang *et al*., 2019) or quinolone resistance genes (Araujo *et al*., 2016; Cavalcanti *et al*., 2015). During infection *P. aeruginosa* also forms biofilms, which are aggregates of cells within an extracellular matrix of exopolysaccharides, extracellular DNA, and proteins. These structures increase both resistance to antibiotics and to the host immune system (Pang *et al*., 2019; Billings *et al*., 2013; Taylor *et al*., 2014).

Due to the multitude of AMR strategies employed by *P. aeruginosa*, treatment with antimicrobial therapy has become significantly less effective across the world (Al‐Orphaly *et al*., 2021). As such, the World Health Organisation (WHO) has now recognised that carbapenem‐resistant *P. aeruginosa* is a global threat to human health (WHO, 2017), highlighting the importance of identifying alternative treatment strategies for *P. aeruginosa* infections, such as phage therapy. The focus of this review is to highlight the challenges posed to phage therapy in light of the recent discovery of lipid renovation in the physiological adaptation of *P. aeruginosa* to phosphorus limitation during infection (Jones *et al*., 2021). Thus, we briefly discuss current understanding of the *P. aeruginosa* lipid membrane as well as the status of *P. aeruginosa* phage research, including the isolation of novel phages and characterisation of their receptors. However, readers are also encouraged to consult excellent recent reviews on AMR mechanisms (Pang *et al*., 2019), cystic fibrosis (Rossi *et al*., 2021; Malhotra *et al*., 2019) and membrane lipids (Kondokova *et al*., 2015; Sohlenkamp and Geiger, 2016), which will only be briefly touched upon where relevant in this review.

## *P. AERUGINOSA* MEMBRANES AND THEIR LIPIDS

**FIGURE 1:**
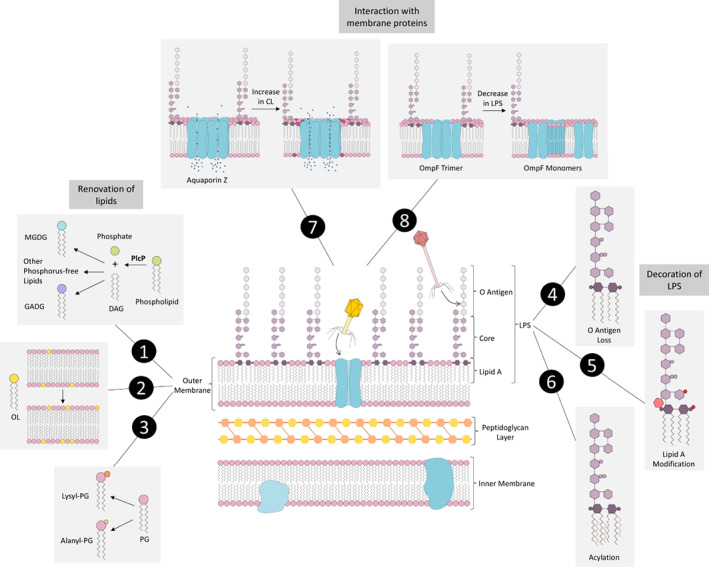
Changes to the outer membrane during *Pseudomonas aeruginosa* infection of its host. The outer membrane is asymmetric with a large proportion of the outer leaflet being made up of LPS. **(1)** In low phosphate conditions the enzyme Phospholipase C (PlcP) removes the phosphate group from phospholipids to leave diacylglycerol (DAG), from which non‐phosphorus‐containing lipids such as MGDG and GADG can be formed (Jones *et al*., 2021). **(2)** In normal growth conditions ornithine lipid (OL) accounts for 2–15% of total lipids, but during low phosphate conditions, or interaction with lung epithelium, the OL level increases (Kim *et al*., 2018). **(3)** *P. aeruginosa* has been shown to modify its membrane lipids through addition of amino acids. PG can be modified with alanine or lysine, which can increase resistance to antimicrobials (Geiger *et al*., 2010; Klein *et al*., 2009). **(4)** *P. aeruginosa* cells from chronic lung infections in people with CF have little or no O antigen on their LPS (Maldonado *et al*., 2016). **(5)** LPS is also modified by addition of the positively charged aminoarabinose and phosphoethanolamine to the lipid A part of the molecule (Edrington *et al*., 2011). **(6)** Acyl chains are also added to lipid A such as the fatty acid palmitate, and secondary acyl chains can be added to the fatty acids. The 3‐position fatty acid may also be removed (Maldonado *et al*., 2016). **(7)** Cardiolipin (CL) stabilises aquaporin Z, a tetrameric water efflux channel, and also increases the transport of water through the channel (Laganowsky *et al*., 2014). **(8)** Outer membrane porin F (OmpF) contains LPS binding sites and forms complexes with LPS molecules. Mutation of one of these sites prevented LPS binding and stopped OmpF forming a trimer (Arunmanee et al., 2016).

The *P*. *aeruginosa* outer membrane, like in other Gram‐negative bacteria, is asymmetric (Figure 1), with the inner leaflet comprising largely glycerophospholipids (GP) and the outer leaflet containing a high concentration of lipopolysaccharide (LPS). LPS has three components: the lipid A anchor, which sits within the membrane, the core oligosaccharide, which is attached to lipid A, and the O oligosaccharide, or O antigen, which is attached to the core oligosaccharide (Needham and Trent, 2013).

The lipid A moiety is made up of hydrophobic acyl chains linked to a backbone glucosamine dimer by ester or amide bonds. The number of acyl chains can vary depending on the species and environmental conditions, but in *P. aeruginosa* there are typically four acyl chains (Maldonado *et al*., 2016). Lipid A is recognised by Toll‐like receptor 4 (TLR4)‐MD2, which triggers an inflammation response in order to try and clear the bacteria (Ciesielska *et al*., 2021). Some *P. aeruginosa* strains have no O antigen in their LPS, and this is known as “rough‐type” LPS (whereas LPS with O antigen is “smooth”) (Maldonado *et al*., 2016). All components of the LPS can undergo modification under different conditions. For example the removal, addition, or modification of phosphate groups on LPS can alter the charge of the membrane, influencing susceptibility to cationic antimicrobial peptides (CAMPs) (Powers and Trent, 2018).

Besides LPS, the main lipids in membranes of *Pseudomonas* species are GP, of which the most common are phosphatidylethanolamine (PE), phosphatidylglycerol (PG), cardiolipin (CL), and phosphatidylcholine (PC) (Table 1) (Kondakova *et al*., 2015). PE and PG make up the vast majority of GP in the membrane, but during stationary phase growth CL can accumulate to up to 10% of all GP (Kondakova *et al*., 2015). Different lipid species have different charges and functions within the membrane (Sohlenkamp and Geiger, 2016). PE, the most abundant GP in *Pseudomonas* membranes is zwitterionic at pH 7 (Sohlenkamp and Geiger, 2016), and is important in maintaining membrane structure by increasing lateral pressure and introducing curvature stress (Kondakova *et al*., 2015). PE is also a precursor to a number of essential biological molecules such as diacyl glycerol (DAG), fatty acids, phosphatidic acid (PA) and LPS (Gibellini and Smith, 2010). At pH 7 PC is also zwitterionic, whereas PG and CL are both anionic. PG can form intermolecular H bonds within the membrane (Zhao *et al*., 2008), which is important for membrane stability. CL is synthesised by the condensation of two PG molecules and plays a role in the formation of dynamic protein‐lipid membrane domains with high curvature, such as at sites of bacterial division (Mileykovskaya and Dowhan, 2005).

**TABLE 1:** Characteristics of major membrane lipids found in *P. aeruginosa*

Lipid	Structure	Charge at pH 7
Phosphatidylethanolamine (PE)	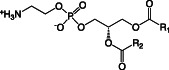	Zwitterionic
Phosphatidylglycerol (PG)	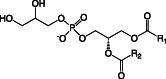	Anionic
Cardiolipin (CL)	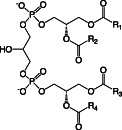	Anionic
Phosphatidylcholine (PC)	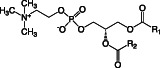	Zwitterionic
Monoglucosyl diacylglycerol (MGDG)	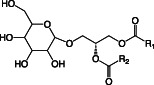	Neutral
Glucuronic acid diacylglycerol (GADG)	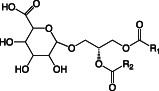	Anionic
Ornithine lipid (OL)	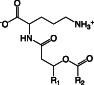	Zwitterionic

Modifications to membrane lipids can occur in response to environmental conditions (Geiger *et al*., 2010; Klein *et al*., 2009). An example of this is the addition of amino acids (Figure 1), such as the addition of lysine to PG to form lysyl‐PG, which has been observed in *P. aeruginosa*, and can increase resistance to CAMPs (Geiger *et al*., 2010). PG can also be modified with alanine in *P. aeruginosa* under acidic growth conditions, which can increase resistance to certain antimicrobials e.g. Cr^3+^, and the osmolyte sodium lactate (Klein *et al*., 2009).

Under low phosphate conditions *P. aeruginosa* upregulates the *olsBA* operon which synthesises the phosphate‐free ornithine lipid (OL) (Lewenza *et al*., 2011; Jones *et al*., 2021). We have recently shown that under phosphorus‐limited conditions *P. aeruginosa* substitutes membrane phospholipids with the non‐phosphorus containing glyceroglycolipids monoglucosyldiacylglycerol (MGDG) and glucuronic acid diacylglycerol (GADG) (Table 1). The synthesis of glyceroglycolipids in *P. aeruginosa* is carried out by a two‐step pathway, involving a metallophospholipase PlcP and two glycosyltransferases, denoted Agt1 and Agt2 (Jones *et al*., 2021). *P. aeruginosa* has been shown to upregulate the *olsBA* operon, *plcP*, *agt1*, and *agt2* upon interaction with human epithelial cells (Frisk *et al*., 2004; Chugani and Greenberg, 2007), and in sputum samples from CF patients *P. aeruginosa* upregulates *olsBA* and *agt2* (Rossi *et al*., 2018). Low phosphate levels can also occur in the serum post‐operatively or following major burns and is associated with worse clinical outcomes (Sadot *et al*., 2019; Loghmani *et al*., 2010). Hypophosphatemia is especially prevalent in intensive care units (Chen *et al*., 2021). Therefore, studying the response of *P. aeruginosa* to low phosphate levels is important to understanding its infection biology.

## ADAPTATION OF *P*. *AERUGINOSA* TO THE CF LUNG ENVIRONMENT

Most new *P. aeruginosa* infections that occur in individuals with CF come from the environment, rather than from the lungs of other infected individuals (Yang *et al*., 2011). On entry to the lung, *P. aeruginosa* needs to adapt to complex and variable micro‐environments in order to survive (Folkesson *et al*., 2012; Garg *et al*., 2017). Pressures encountered include osmotic stress due to the presence of high viscosity mucus, varying oxygen levels, and reactive oxygen and nitrogen species produced by the inflammatory response (Winstanley *et al*., 2016; Sommer *et al*., 2020; Bhagirath *et al*., 2016). CF patients will also likely be being treated long term with a variety of antibiotics (Winstanley *et al*., 2016), which by definition presents a challenge to *P. aeruginosa*. Bacterial adaptation involves several key strategies, including modification of lipid A, changes in membrane lipid composition and the formation of biofilms (Needham and Trent, 2013; Frisk *et al*., 2004; Yang *et al*., 2011).

Soon after colonisation of the CF airway, lipid A of *P. aeruginosa* is modified through both the addition of the positively charged amino sugar residue aminoarabinose and the addition of palmitate (Figure 1) (Ernst *et al*., 2007). Aminoarabinose alters the charge of lipid A, increasing resistance to innate immune elements including CAMPs and complement. The addition of the fatty acid palmitate (Figure 1) is catalysed by the PagP enzyme which is under the control of the PhoPQ regulatory system (Thaipisuttikul *et al*., 2014). The addition of palmitate increases membrane integrity and decreases TLR4 activation (Needham and Trent, 2013). Another modification under the control of PhoPQ is the removal of the 3‐position fatty acid by PagL, which has been shown to occur in *P. aeruginosa* (Ernst *et al*., 2006). Under‐acylation of lipid A is known to lower the inflammatory response (Di Lorenzo *et al*., 2015). *P. aeruginosa* can also add secondary acyl chains to the fatty acids of lipid A (Maldonado *et al*., 2016).

*P. aeruginosa* cells in the early stage of infection have complete O antigens, but those from chronic lung infections in CF patients have a rough LPS phenotype, with short or no O‐antigens (Figure 1) (Maldonado *et al*., 2016). Loss of the O‐antigen seems to confer an advantage as it is immunogenic, and therefore cells lacking it will be less likely to be detected and destroyed by the immune system. However, cells lacking the O‐antigen are also less virulent (Kintz and Goldberg, 2008).

Adapting to the CF lung environment also involves changes to membrane lipids (Naughton *et al*., 2011; Frisk *et al*., 2004). The cardiolipin synthase gene has been shown to be upregulated in *P. aeruginosa* during chronic infection of the CF lung (Naughton *et al*., 2011), indicating increased proportions of CL in the membrane. Addition of amino acids to phospholipids may also occur during infection (see section 2), and can increase resistance to CAMPs, bacteriocins, and antibiotics.

Another lipid which is altered in infection is OL. In low phosphate environments (e.g Gao *et al*., 2004), or on interaction with the lung epithelium (Frisk *et al*., 2004; Chugani and Greenberg, 2007), the percentage of OLs in the membrane massively increases (Figure 1). With increased levels of OLs the charge and hydrophobicity of the membrane changes and the susceptibility of *P. aeruginosa* to CAMPs and antibiotics is reduced. Binding of macrophages is lessened, and biofilm formation is enhanced (Kim *et al*., 2018). It has been shown that OLs decrease the expression of two macrophage enzymes involved in inflammation (Kim *et al*., 2018). Together, this suggests that increased OL contributes to the persistence of *P. aeruginosa* in chronic infection.

Interestingly, our own recent data suggests that membrane lipid renovation in *P. aeruginosa* in response to phosphorus stress during lung infection also confers elevated resistance to antimicrobial peptides. Indeed, these surrogate glyceroglycolipids increase resistance to polymyxin B in *P. aeruginosa* as well as recombinant *Escherichia coli* strains overexpressing glyceroglycolipids (Jones *et al*., 2021). This newly discovered lipid renovation strategy could potentially play an important role in the adaptation of *P. aeruginosa* during lung infection. Analysis of metatranscriptomic datasets from sputum samples taken from CF patients showed overproduction of PlcP and Agt, two of the key enzymes responsible for the formation of the glyceroglycolipids MGDG and GADG (Figure 1) (Jones *et al*., 2021), as well as alkaline phosphatase PhoA, suggesting *P. aeruginosa* is experiencing phosphorus limitation during lung infection.

In the CF lung, biofilm formation can also provide protection for *P. aeruginosa* against phagocytosis, antibiotics, antibodies, osmotic stress and oxidative stress (Yang *et al*., 2011). Accumulation of mutations which lead to mucoidy and biofilm formation are seen in *P. aeruginosa* in CF patients (Ernst *et al*., 2006), alongside the upregulation of genes important for biofilm formation (Rossi *et al*., 2020). When biofilms initially form they have a higher diversity of fatty acids in their phospholipids than planktonic cells, and the amount of branched fatty acid chains is increased. However, as the biofilm ages, the diversity decreases again, and they become more like planktonic bacteria again (Benamara *et al*., 2014). This may be because the biofilm cells are preparing to become planktonic again to seek out a new place to form a biofilm.

The chronic use of antibiotics in patients with CF may also impact the membrane lipids of *P. aeruginosa*. Cationic peptides can cause clustering of anionic lipids in the membrane, with CL segregating into domains in the presence of CAMPS (Epand *et al*., 2016). Polymyxin B, an antibiotic of last resort against *P. aeruginosa*, has been shown to cause lipid exchange between the inner and outer membrane of Gram‐negative bacteria (Berglund *et al*., 2015; Clausell *et al*., 2007; Yu *et al*., 2015). Polymyxins (Polymyxin B and Colistin) work by binding to LPS and disrupting the outer membrane, therefore resistance to them usually involves modification of LPS (Mohapatra *et al*., 2021). The lipid A moieties of polymyxin‐resistant *P. aeruginosa* are modified with aminoarabinose, and the membrane lipid profiles are significantly different from the wild type (Han *et al*., 2018).

## PHAGES INFECTING *PSEUDOMONAS AERUGINOSA* AND PHAGE THERAPY

Bacteriophages (or phages) are viruses that infect bacteria, and can be found in any natural environment where bacteria are found (Dion *et al*., 2020). Phages infecting *Pseudomonas* are widely obtained with ~780 phage isolated and sequenced to date (May, 2022) (Cook *et al*., 2021). They span the diversity of known phage types from ssDNA phages (*Invovidiae*) and RNA phages (*Leviviridae*) to the more common dsDNA phages (*Myoviridae, Podoviridae, Siphoviridae, Autographviridae, Ackermannviridae*). Trials in animals have shown positive outcomes from using phage therapy against *P. aeruginosa* infections. Mouse (Waters *et al*., 2017; Morello *et al*., 2011) and zebrafish (Cafora *et al*., 2019) models of cystic fibrosis have shown significantly improved survival rates and reduced bacterial load, as have mouse models of other forms of *P. aeruginosa* infection (McVay *et al*., 2007; Watanabe *et al*., 2007). A strength of phage therapy is its ability to target and significantly clear *P. aeruginosa* biofilm biomass (Fong *et al*., 2017; Waters *et al*., 2017), where antibiotics largely fail. In rats, phages have been shown to have a synergistic relationship with the antibiotic ciprofloxacin, resulting in 10,000 times more bacterial clearance than either treatment alone (Oechslin *et al*., 2017). Human case reports have demonstrated clearance of *P. aeruginosa* using phage therapy in urinary tract infections, lung infections, infection of an aortic graft, bacteraemia, and more (see Table 2). Wright *et al*., 2009 demonstrated the safety and efficacy of phage therapy for ear infections caused by *P. aeruginosa* in a randomised, double‐blind, placebo‐controlled phase I/II clinical trial (Wright *et al*., 2009). In another such trial, Jault *et al*., 2019 investigated a phage cocktail to treat burn wounds infected with *P. aeruginosa*. However, due to a drop in phage titre after manufacturing, patients were given the cocktail at 4–5 orders of magnitude lower than intended, which did not show efficacy (Jault *et al*., 2019). Recent trials and case studies of the use of phage therapy to treat *P. aeruginosa* infections in humans are summarised in Table 2.

**TABLE 2:** Phage therapy trials for *P. aeruginosa* infection

Phase	Infection site	Phage used	In conjunction with antibiotics?	Background	Result	Reference
Case report	Urinary tract	Eliava Institute Pyophage #051007	Yes, meropenem and colistin	Previous treatment with antibiotics alone had not been successful.	No *P. aeruginosa* detected after 10 days treatment. None detected a year later.	Khawaldeh *et al*., 2011
Case report	Aortic graft/blood (bacteraemia)	Phage OMKO1	Yes, ceftazidime	Repeated infections after an aortic graft over the course of 3 years despite antibiotic use.	Cultures taken from aortic graft showed no *P. aeruginosa*, patient has not had any repeat infections.	Chan *et al*., 2018
Case report	Heart/blood (bacteraemia)	Two phages which exhibited lytic activity against the patient's isolate	Yes, meropenem, tobramycin and polymyxin B	Infant with multiple health conditions experienced *P. aeruginosa* infection after an operation on the heart. Patient was allergic to multiple antibiotic categories.	Blood cultures after phage therapy were sterile, but infection returned on cessation of phage administration.	Duplessis *et al*., 2018
Phase I/II trial	Ear	Six phages (BC‐BP‐01 to BC‐BP‐06,15 NCIMB deposit numbers 41,174–41,179)	No	Randomised, double‐blind, placebo‐controlled Phase I/II clinical trial in 24 patients with antibiotic‐resistant ear infections.	*P. aeruginosa* counts significantly lower in the phage treated group along with significant improvement in clinical indicators.	Wright *et al*., 2009
Phase I/II trial	Burn wound	A cocktail of 12 natural lytic anti‐*P. aeruginosa* bacteriophages	No	Randomised, controlled, double‐blind Phase I/II clinical trial with 25 patients with infected burn wounds.	Phage reduced bacterial counts, but less than the standard treatment. Phage titres dropped after manufacturing, giving a daily dose of 10–100 PFU/mL, around 5–6 log lower than intended. This may be the reason for the lack of success with the phage.	Jault *et al*., 2019
Case report	CF lung	Pyophage preparation administered by nebulizer	Partially, tetracycline	Child with CF with a *P. aeruginosa* and *S*. *aureus* infection. Infection had not responded to other treatment. Bacteria became more sensitive to certain antibiotics after phage treatment.	After 3 rounds of phage therapy, the final one in conjunction with tetracycline, no *P. aeruginosa* or *Staphylococcus aureus* could be found in sputum. Phage therapy is repeated dependent on pathogen levels.	Kutateladze and Adamia, 2008
Case report	Burn wound	Not stated	Yes, ceftazidime	A patient with *P. aeruginosa*‐infected burn wounds which did not respond to antibiotics.	After 3 days, no *P. aeruginosa* could be isolated from wounds. Unclear whether phage, antibiotics, or the combination was responsible for the improvement.	Marza *et al*., 2006
Phase I trial	Venous leg ulcers	WPP‐201 cocktail	No	39 patients with ulcers were treated for 12 weeks with either phage or a saline control.	No adverse events recorded. No significant difference in frequency of adverse events, or in rate or frequency of healing.	Rhoads *et al*., 2009
Case report	Wound, blood (bacteraemia)	BFC1 cocktail	No	Wound colonised with multidrug resistant *P. aeruginosa*, leading to colistin‐only‐sensitive *P. aeruginosa* septicaemia. Patient developed acute kidney injury due to the infection and colistin, so therapy was stopped. Patient went into a coma and was treated with intravenous phage as all other treatment options had ran out.	Immediately blood cultures became negative, and fever disappeared. After a few days kidney function recovered. Wounds remained infected which caused further episodes of sepsis which were treated with antibiotics.	Jennes *et al*., 2017
Case report	CF lung	AB‐PA01 cocktail	Yes, azithromycin, ciprofloxacin, colistin, doripenem, linezolid, piperacillin‐tazobactam and vancomycin	Patient developed multidrug resistant *P. aeruginosa* pneumonia, and colistin‐induced renal failure. The infection was not responding to antibiotics.	Pneumonia clinically resolved, colistin was discontinued, there was a return to baseline renal function, supplemental oxygen requirements were reduced, and there was no recurrence of *P. aeruginosa* pneumonia or CF exacerbation within 100 days of treatment. Patient underwent successful bilateral lung transplant 9 months later.	Law *et al*., 2019
Case report	Knee prosthesis and femur	Not stated	Yes, gentamicin, clindamycin, colistin, meropenem and ceftazidime	Extensively drug resistant *P. aeruginosa* isolated from knee prosthesis.	No *P. aeruginosa* detected on days 3, 4, or 5 of phage treatment. 10 months later there were no signs of infection.	Tkhilaishvili *et al*., 2019
Case report	Lung	AB‐PA01 and Navy phage cocktails	Yes, piperacillin‐tazobactam, tobramycin and colistin	2 lung transplant recipients with multidrug‐resistant *P. aeruginosa*.	Clinical improvement seen in both patients compared to antibiotics alone. *P. aeruginosa* did return in both patients, but in patient two did not return for two months. In both cases isolates showed increased susceptibility to several antibiotic classes.	Aslam *et al*., 2019
Case report	Heart	PA5, PA10	No	Prosthetic infection after aortic arch replacement. Many bacterial species, including *P. aeruginosa*. Conventional antibiotic therapy had not been successful.	Bacteria no longer detected and phage therapy was stopped. 17 days later developed *P. aeruginosa* and *E. coli* infection. However, second *P. aeruginosa* may have been independent of the first infection.	Rubalskii *et al*., 2020
Case report	Wound	PA5, PA10	No	*P. aeruginosa* infection of sternal wound abscesses after double lung transplant. Conventional antibiotic therapy had not been successful.	Wound healed and no *P. aeruginosa* could be detected.	Rubalskii *et al*., 2020

The studies in this Table were found by searching “*Pseudomonas* phage trial” in Pubmed for the last 15 years and all the results that described using phage as a therapy for *P. aeruginosa* infection were included.

In order to infect a bacterium, a phage first binds to its receptor on the bacterial cell surface. The receptor is often a type of lipid‐anchored polysaccharide or a membrane protein (Chaturongakul and Ounjai, 2014). Some phages are extremely specific to a single species or strain of bacteria, while others can have a broader host range, and hence the receptor plays an important role in dictating the host range of the phage (de Jonge *et al*., 2019). While some mechanisms of phage binding are well‐characterised e.g. the binding of phage T4 to *E. coli* (Brzozowska *et al*., 2018; Washizaki *et al*., 2016), there is much that is still not known. One particular aspect is how phage binding is affected by the altered membrane lipid composition of its host, if/when this occurs during the infection process, which may alter the orientation, structure or function of a phage receptor. We consider this next with specific regard to *P. aeruginosa* infections and the development of effective phage therapy strategies.

**FIGURE 2:**
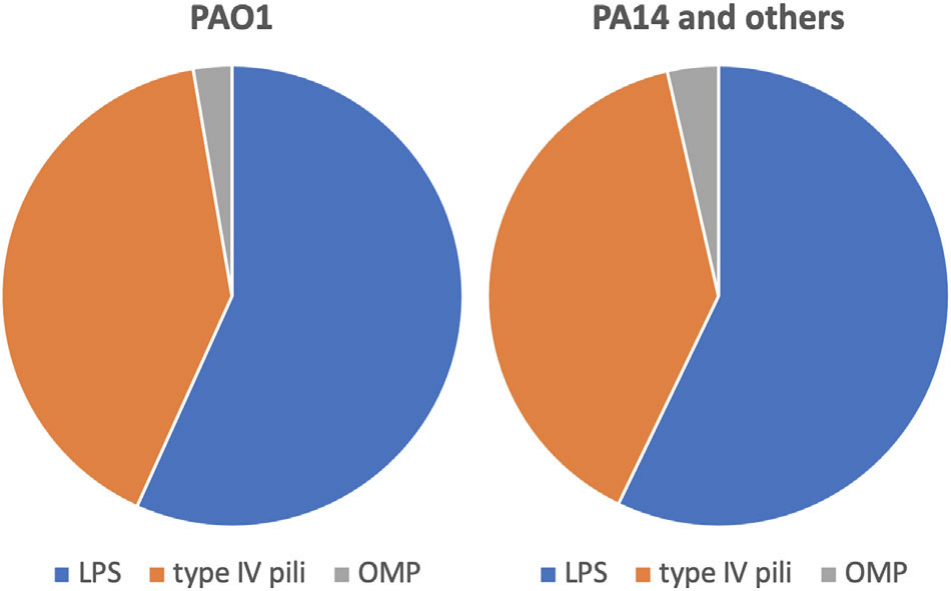
Studies showing known phage receptors in *P. aeruginosa* strain PAO1 (left panel, n = 36), PA14 and others (right panel, n = 27). LPS, lipopolysaccharides; OMP, outer membrane proteins. Studies used in this analysis are shown in Supplementary Table 1

There are at least 50 *P. aeruginosa* phages which have had their receptors characterised (Figure 2; Supplementary Table 1). Most of the *P. aeruginosa* phage receptors are either the type IV pilus or LPS, but at least one phage uses outer membrane porin M (OprM) as its receptor (Chan *et al*., 2016). There are undoubtedly far more membrane proteins and structures that are used as phage receptors on the surface of *P. aeruginosa*, as only a small proportion of known *P. aeruginosa* phages have a receptor identified.

## THE IMPACT OF THE LIPID ENVIRONMENT ON PHAGE RECEPTORS AND PHAGE THERAPY

As phage receptors are either membrane proteins, polysaccharides or other membrane structures (Silva *et al*., 2016), their presence will most likely be influenced by their lipid surroundings. The changes to lipids that occur in *P. aeruginosa* during adaptation to the CF lung environment, described earlier, including LPS modifications and the production of non‐phosphate lipid classes, are very likely to have an impact on membrane properties. As detailed below, the lipid environment of membrane proteins has an important influence on their structure and function. Notably, one major mechanism of phage resistance is through changes to phage receptors, such as masking, removing or modifying them (Olszak *et al*., 2017). This highlights how even small changes in the receptor can prevent phage binding, and therefore phage infection.

There are certainly precedents that changes in membrane lipids can affect their interaction with membrane proteins (for a summary, see Table 3). For example, the membrane protein aquaporin Z (AqpZ), a tetrameric water efflux channel in *E. coli*, was shown to be stabilised by membrane lipids, especially by CL (Figure 1). Further experiments showed that CL modulates the function of ApqZ (Laganowsky *et al*., 2014). The lipid phosphatidylinositol was found to bind to and stabilise the mechanosensitive channel of large conductance (MscL) greater than other lipids, although all lipids tested stabilised MscL to some extent (Laganowsky *et al*., 2014). AmtB, a trimeric *E. coli* ammonia channel, is stabilised by CL and PG. Stabilisation of the protein increases linearly with the amount of these lipids added. Further investigation showed that AmtB has selectivity for PG‐like head groups. AmtB contains a loop which forms a lipid‐binding site with PG or CL, through hydrogen bonds, a water bridge and hydrophobic interactions (Laganowsky *et al*., 2014). The translocon is a complex of proteins which translocate proteins across the inner membrane of Gram‐negative bacteria. It includes transmembrane proteins SecYEG, and cytoplasmic protein SecA, which binds to SecYEG in the process of translocation (Ryabichko *et al*., 2020). SecYEG requires CL for its stability and efficient function as a high affinity binder of SecA. The interaction of the positively charged N‐terminus of SecA with negatively charged membrane lipids primes it allosterically for binding to SecYEG (Ryabichko *et al*., 2020). PG binds to outer membrane porin F (OmpF), which is made up of three pore channels, and stabilises it in an open conformation. Binding also promotes the opening of closed pores and enhances ion transport activity. OmpF preferentially binds PG over zwitterionic lipids, such as PC (Liko *et al*., 2018). OmpF is conserved as a phage receptor across many different bacterial hosts and phage types, including phage Yep‐phi (family *Autographiviridae,* genus *Berlinvirus)* that infects *Yersinia pestis* (Zhao *et al*., 2013), phage vB_YenM_TG1 (family *Straboviridae,* genus *Tegunvirus)* that infects *Yersinia enterocolitica,* phages fPS‐2 and fPS‐90 (family *Straboviridae*, genus *Tequatrovirus*) that infect *Yersinia pseudotuberculosis*, phage T2 (family *Straboviridae*, genus *Tequatrovirus*) and vB_EcoM_IME281 (family *Straboviridae*, genus *Dhakavirus)* that infect *Escherichia*. It is therefore possible that changes to the levels of PG in the outer membrane could cause conformational changes in OmpF, altering phage adsorption. OmpF has also been shown to contain a number of LPS binding sites, forming complexes with a variable number of LPS molecules (Figure 1). Mutation of one of these binding sites prevented LPS binding and stopped OmpF forming a trimer *in vivo* (Arunmanee et al., 2016). Interactions between the LPS layer and membrane proteins are likely to be important in the impermeability of the outer membrane (Arunmanee et al., 2016).

**TABLE 3:** Summary of examples of membrane lipids and lipopolysaccharides interacting with membrane proteins

Lipid	Membrane Protein	Nature of Interaction	Bacteria Observed in	Reference
CL	Aquaporin Z	Stabilise and modulate function	*E. coli*	Laganowsky *et al*., 2014
CL	Ammonium channel AmtB	Stabilise	*E. coli*	Laganowsky *et al*., 2014
CL	SecYEG of translocon complex	Stabilise and modulate function	*E. coli*	Ryabichko *et al*., 2020
PG; LPS	Outer membrane porin F (OmpF)	Stabilise in open conformation, enhance ion transport activity; strong interaction with LPS	*E. coli*	Liko *et al*., 2018; Arunmanee et al., 2016
PG	Ammonium channel AmtB	Stabilise	*E. coli*	Laganowsky *et al*., 2014
PI	Mechanosensitive channel of large conductance	Stabilise	*E. coli*	Laganowsky *et al*., 2014
Anionic lipids	Potassium channel KcsA	Important in potassium‐conducting function	*Streptomyces lividans*	Contreras *et al*., 2011
LPS	Membrane porin OprH	OprH shows strong interaction with LPS	*P. aeruginosa*	See Supplementary Table 1

The potassium channel KcsA selects for anionic lipids in its core, and these lipids are important for the potassium‐conducting function of the protein (Contreras *et al*., 2011). The interactions between negatively charged phospholipids and positively charged amino acids may help to guide the orientation of membrane proteins (Contreras *et al*., 2011). An example of this is the interaction of lactose permease (LacY) with the anionic lipids PG and CL in *E. coli*. The N‐terminal helical bundle of LacY can be completely inverted, have a mixed topology, or a fully native topology as the percentage of the zwitterionic PE in the membrane compared to the anionic PG and CL is increased from 0% to 70%. This change in conformation happens due to changes in lipid ratios both at the time of LacY insertion into the membrane, and after insertion (Vitrac *et al*., 2013).

The presence of aminoacyl phospholipids may also affect the rigidity, fluidity and permeability of the membrane. The presence of aminoacyl phospholipids in vesicles has been shown to stabilise the bilayer and alter peptide binding behaviour (Slavetinsky *et al*., 2017). Ordinarily, magnesium cations bridge adjacent LPS molecules. Under magnesium‐deficient growth conditions, outer membrane protein H (OprH) is upregulated and becomes a major part of the *P. aeruginosa* outer membrane (Edrington *et al*., 2011). OprH contains multiple LPS interaction sites allowing it to interact with multiple LPS molecules at once and facilitating the formation of cross‐links. In turn, these cross‐linkages between LPS molecules increase its stability and decrease membrane permeability (Edrington *et al*., 2011). OprH is genetically linked to the PhoPQ regulatory system, where the two‐component system is upregulated in response to magnesium deficiency. LPS alterations, such as the aminoarabinose and palmitate additions, are also regulated by the PhoPQ system. OprH may have a higher affinity for LPS when these modifications are present (Edrington *et al*., 2011).

These examples demonstrate the importance of membrane lipids in the structure and function of membrane proteins and LPS, both of which can be receptors for *P. aeruginosa* phages. Therefore, when *P. aeruginosa* undergoes lipid renovation in response to phosphorus limitation during infection, this will likely have an impact on membrane properties. Comparing the proteomes of wild‐type *P. aeruginosa* and a *plcP*‐deletion mutant, both under phosphorus limitation, revealed several membrane proteins to be differentially expressed depending on the ability to remodel lipids (Jones *et al*., 2021). This included PilC, a membrane porin (OpdP), and an outer membrane receptor (FptA) (Jones *et al*., 2021). From these data it is thus conceivable that changes in membrane lipids may have subsequent knock‐on effects for phage therapy. This could be due to an impact on general membrane properties following lipid remodelling, or due to direct interactions with a particular protein. As a proof‐of‐concept, we have observed that phage adsorption efficiency can indeed be affected by lipid remodelling in response to phosphorus limitation (R. Lyon unpublished data).

In conclusion, we argue that it is important to better understand the relationship between the environment, the bacterial cell surface and the subsequent impact on phage receptors and phage absorption. This further links to how changes in membrane lipid composition in response to phosphorus limitation during lung infections may affect the efficacy of phage therapy. Certainly, problems have been encountered translating the success of phage therapy in the laboratory to success in clinical trials (Valente *et al*., 2021). While this is likely to be the result of many confounding factors, one of these could be the influence of the changing lipid makeup of the bacterial membrane and its subsequent impact on phage receptors. We propose that considering both the native lipid environment and the lipid remodelled membrane while developing a phage cocktail will be important in increasing the likelihood of success of phage therapy when treating *P. aeruginosa* lung infections.

## Supporting information


**Supplementary Table 1** *Pseudomonas aeruginosa* phages and their receptors
