# Supplementary material for: Membrane lipid renovation in Pseudomonas aeruginosa ‐ implications for phage therapy?
Source: Environ Microbiol. 2022 Aug 14;24(10):4533–46. doi: 10.1111/1462-2920.16136 (PMC9804370; doi:10.1111/1462-2920.16136)
Supplement: Supplementary file 1 — Supplementary Table 1 Pseudomonas aeruginosa phages and their receptors [file EMI-24-4533-s001.docx]

**Supplementary Table 1. *Pseudomonas aeruginosa* phages and their receptors**

| **Strain** | **Phage** | **Receptor** | **Family*** | **Genus** | **References** |
| --- | --- | --- | --- | --- | --- |
| PAO1 | vB_PaeA_QDWS | LPS | *Autographiviridae* |  | Xuan *et al.*, 2022 |
| PAO1 | vB_PaeM_JG004 | LPS | Formerly *Myoviridae* | *Pakpunavirus* | Garbe *et al.*, 2011 |
| PAO1 | vB_PaeM_PIK | LPS | *Formerly Myoviridae* |  | Patel and Rao, 1983 |
| PAO1 | vB_PaeM_Ab17 | LPS | *Formerly Myoviridae* |  | Pourcel *et al.*, 2020 |
| PAO1 | vB_PaeA_LKA1 | LPS | *Autographiviridae* | *Stubburvirus* | Ceyssens *et al.*, 2006 |
| PAO1 | vB_Pae_PA10P2 | LPS |  |  | Wright *et al.*, 2019 |
| PAO1 | vB_PaeM_14/1 | LPS | Formerly *Myoviridae* |  | Wright *et al.*, 2019 |
| PAO1 | vB_PaeP_PEV2 | LPS | *Schitoviridae* | *Litunavirus* | Shiley *et al.*, 2017 |
| PAO1 | vB_PaeM_KT28 | LPS | Formerly *Myoviridae* | *Pbunavirus* | Danis-Wlodarczyk *et al.*, 2015 |
| PAO1 | vB_PaeM_KTN6 | LPS | Formerly *Myoviridae* | *Pbunavirus* | Danis-Wlodarczyk *et al.*, 2015 |
| PAO1 | vB_PaeM_φPA01 | LPS | Formerly *Myoviridae* | *Pbunavirus* | Ong *et al.*, 2020 |
| PAO1 | vB_PaeM_φPA02 | LPS | Formerly *Myoviridae* | *Phikzvirus* | Ong *et al.*, 2020 |
| PAO1 | vB_PaeP_φIBB-PAA2 | LPS | Formerly *Podoviridae* | *Bruynoghevirus* | Pires *et al.*, 2017 |
| PAO1 | vB_PaeM_CEB_DP1 | LPS | Formerly *Myoviridae* | *Pbunavirus* | Pires *et al.*, 2017 |
| PAO1 | vB_PaeM_JG024 | LPS | Formerly *Myoviridae* | *Pbunavirus* | Garbe *et al.*, 2010 |
| PAO1 | vB_PaeC_φYY | LPS core | *Cystoviridae* |  | Yang *et al.*, 2020 |
| PAO1 | vB_PaeM_PaoP5 | LPS O antigen | Formerly *Myoviridae* | *Pakpunavirus* | Yang *et al.*, 2020 |
| PAO1 | vB_PaeM_MPK1 | LPS O antigen | *Formerly Myoviridae* |  | Heo *et al.*, 2009 |
| PAO1 | vB_PaeA_MPK6 | LPS O antigen | *Autographiviridae* | *Phikmvvirus* | Heo *et al.*, 2009 |
| PAO1 | vB_Pae_FIZ15 | LPS O antigen |  |  | Vaca-Pacheco *et al.*, 1999 |
| PAO1 | vB_PaeM_PA5oct | LPS and type IV pili | Formerly *Myoviridae* |  | Olszak *et al.*, 2019 |
| PAO1 | vB_PaeA_MPK7 | Type IV pili | *Autographiviridae* | *Phikmvvirus* | Bae and Cho, 2013 |
| PAO1 | vB_PaeS_JBD26 | Type IV pili | Formerly *Siphoviridae* | *Casadabanvirus* | Harvey *et al.*, 2018 |
| PAO1 | vB_PaeS_JBD68 | Type IV pili | Formerly *Siphoviridae* |  | Harvey *et al.*, 2018 |
| PAO1 | vB_PaeS_DLP1 | Type IV pili | Formerly *Siphoviridae* |  | McCutcheon *et al.*, 2018 |
| PAO1 | vB_PaeS_DLP2 | Type IV pili | Formerly *Siphoviridae* |  | McCutcheon *et al.*, 2018 |
| PAO1 | vB_Pae_PA5P2 | Type IV pili |  |  | Wright *et al.*, 2019 |
| PAO1 | vB_PaeL_PP7 | Type IV pili | *Leviviridae* | *Unclassified* | Kim *et al.*, 2018a |
| PAO1 | vB_PaeA_LUZ19 | Type IV pili | *Autographiviridae* | *Phikmvvirus* | Lavigne *et al.*, 2013 |
| PAO1 | vB_PaeS_B3 | Type IV pili | Formerly *Siphoviridae* | *Beetrevirus* | Roncero *et al.*, 1990 |
| PAO1 | vB_PaeS_D3112 | Type IV pili | Formerly *Siphoviridae* | *Casadabanvirus* | Roncero *et al.*, 1990 |
| PAO1 | vB_Pae_PT7 | Type IV pili |  |  | Wright *et al.*, 2019 |
| PAO1 | vB_PaeM_φKZ | Type IV pili | Formerly *Myoviridae* | *Phikzvirus* | Hertveldt *et al.*, 2005 |
| PAO1 | vB_PaeS_PA1Ø | Type IV pili | Formerly *Siphoviridae* |  | Kim *et al.*, 2012 |
| PAO1 | vB_PaeM_KTN4 | Type IV pili | *Formerly Myoviridae* | *Phikzvirus* | Danis-Wlodarczyk *et al.*, 2016 |
| PAO1 | vB_PaeM_OMKO1 | OprM | Formerly *Myoviridae* |  | Chan *et al.*, 2016 |
| PA14 | vB_PaeM_LMA2 | LPS | Formerly *Myoviridae* | *Pbunavirus* | Alseth *et al.*, 2019 |
| PA14 | vB_PaeS_Ab30 | Type IV pili | *Formerly Myoviridae* |  | Pourcel *et al.*, 2020 |
| PA14 | vB_PaeS_MP22 | Type IV pili | Formerly *Siphoviridae* | *Casadabanvirus* | Heo *et al.*, 2007 |
| PA14 | vB_PaeS_DMS3 | Type IV pili | Formerly *Siphoviridae* | *Casadabanvirus* | Budzik *et al.*, 2004 |
| PA14 | vB_PaeS_DMS3 | Type IV pili | Formerly *Siphoviridae* | *Casadabanvirus* | Alseth *et al.*, 2019 |
| PcyII-10 | vB_PaeP_C2-10_Ab09 | LPS | *Schitoviridae* | *Litunavirus* | Pourcel *et al.*, 2017 |
| PcyII-10 | vB_PaeM_Ab27 | LPS | Formerly *Myoviridae* | *Pbunavirus* | Pourcel *et al.*, 2017 |
| PcyII-10 | vB_PaeL_LeviOr01 | Type IV pili | *Leviviridae* |  | Pourcel *et al.*, 2017 |
| PcyII-10 | vB_PaeA_PAO1_Ab05 | Type IV pili | *Autographiviridae* | *Phikmvvirus* | Pourcel *et al.*, 2017 |
| PcyII-10 | vB_PaeP_Ab12 | Type IV pili | Formerly *Podoviridae* |  | Pourcel *et al.*, 2017 |
| PcyII-10 | vB_PaeS_SCH_Ab26 | Type IV pili | Formerly *Siphoviridae* | *Septimatrevirus* | Pourcel *et al.*, 2017 |
| PcyII-10 | vB_PaeP_φC725A | Type IV pili | Formerly *Podoviridae* | *Hollowayvirus* | Pourcel *et al.*, 2017 |
| PcyII-10 | vB_PaeS_pfII40A | Type IV pili | Formerly *Siphoviridae* | *Casadabanvirus* | Pourcel *et al.*, 2017 |
| PAK | vB_PaeM_K5 | LPS | Formerly *Myoviridae* | *Pakpunavirus* | Li *et al.*, 2016 |
| PAK | vB_PaeP_O4 | LPS O antigen | Formerly *Podoviridae* |  | Zhang *et al.*, 2018 |
| PAK | vB_PaeM_K8 | LPS O antigen | Formerly *Myoviridae* | *Pakpunavirus* | Pan *et al.*, 2016 |
| AK1401 | vB_PaeS_A7 | LPS | Formerly *Siphoviridae* |  | Rivera *et al.*, 1992 |
| PA1 | vB_PaeM_PaP1 | LPS | Formerly *Myoviridae* | *Pakpunavirus* | Le *et al.*, 2013 |
| NCTC 8505 | vB_Pae_H22 | LPS |  |  | Temple *et al.*, 1986 |
| C2-10 | vB_PaeM_Ab01 | LPS | *Formerly Myoviridae* |  | Pourcel *et al.*, 2020 |
| US449 | vB_PaeP_LUZ7 | LPS | Formerly *Podoviridae* | *Luzseptimavirus* | Ceyssens *et al.*, 2010 |
| PAC1 | vB_PaeM_E79 | LPS core | Formerly *Myoviridae* | *Pbunavirus* | Meadow and Wells, 1978 |
| PML14 | vB_PaeM_φCTX | LPS core | *Peduoviridae* | *Citexvirus* | Yokota *et al.*,1994 |
| AK-43 and AK-44 | vB_PaeP_φPLS27 | LPS core | Formerly *Podoviridae* |  | Jarrell and Kropinski, 1981 |
| BI | vB_PaeS_Phage 2 | LPS O antigen | Formerly *Siphoviridae* |  | Bartell *et al.*, 1971 |
| Li010 | vB_PaeP_LUZ24 | Type IV pili and LPS | Formerly *Podoviridae* | *Bruynoghevirus* | Ceyssens *et al.*, 2008 |
| PA16 | vB_PaeM_PIAS | OrpM-MexXY | Formerly *Myoviridae* | *Pakpunavirus* | Valappil *et al.*, 2021 |

To find the phage for this Table the term “*Pseudomonas* phage receptor” was searched in Pubmed for all years and any phage receptors for *P. aeruginosa* mentioned in the resulting publications were included in the Table. *with the recent update to phage taxonomy the families *Myoviridae, Siphoviridae and Podoviridae* have been disbanded, resulting in many phages not being classified at the family level. For backwards compatibility we have stated their previous family designation, if not assigned to a new family.

**References**

Alseth, E. O., Pursey, E., Luján, A. M., McLeod, I., Rollie, C. and Westra, E. R. (2019). Bacterial biodiversity drives the evolution of CRISPR-based phage resistance. *Nature*, **574**: 549-552.

Bae, H. W. and Cho, Y. H. (2013) Complete genome sequence of *Pseudomonas aeruginosa* podophage MPK7, which requires type IV pili for infection. *Genome Announ*c **1**: e00744-13.

Bartell, P. F., Orr, T. E., Reese, J. F. and Imaeda, T. (1971) Interaction of *Pseudomonas* bacteriophage 2 with the slime polysaccharide and lipopolysaccharide of *Pseudomonas aeruginosa* strain B1. *J Virol* **8**: 311-317.

Budzik, J. M., Rosche, W. A., Rietsch, A. and O'Toole, G. A. (2004) Isolation and characterization of a generalized transducing phage for *Pseudomonas aeruginosa* strains PAO1 and PA14. *J Bacteriol* **186**: 3270-3273.

Chan, B. K., Sistrom, M., Wertz, J. E., Kortright, K. E., Narayan, D. and Turner, P. E. (2016) Phage selection restores antibiotic sensitivity in MDR *Pseudomonas aeruginosa*. *Sci Rep* **6**: a26717.

Ceyssens, P. J., Brabban, A., Rogge, L., Lewis, M. S., Pickard, D., Goulding, D., Dougan, G., Noben, J. P., Kropinski, A., Kutter, E. and Lavigne, R. (2010) Molecular and physiological analysis of three *Pseudomonas aeruginosa* phages belonging to the "N4-like viruses". *Virology* **405**: 26-30.

Ceyssens, P. J., Hertveldt, K., Ackermann, H. W., Noben, J. P., Demeke, M., Volckaert, G. and Lavigne, R. (2008) The intron-containing genome of the lytic *Pseudomonas* phage LUZ24 resembles the temperate phage PaP3. *Virology* **377**: 233-238.

Ceyssens, P. J., Lavigne, R., Mattheus, W., Chibeu, A., Hertveldt, K., Mast, J., Robben, J. and Volckaert, G. (2006) Genomic analysis of *Pseudomonas aeruginosa* phages LKD16 and LKA1: establishment of the phiKMV subgroup within the T7 supergroup. *J Bacteriol* **188**: 6924-6931.

Danis-Wlodarczyk, K., Olszak, T., Arabski, M., Wasik, S., Majkowska-Skrobek, G., Augustyniak, D., Gula, G., Briers, Y., Jang, H. B., Vandenheuvel, D., Duda, K. A., Lavigne, R. and Drulis-Kawa, Z. (2015) Characterization of the newly isolated lytic bacteriophages KTN6 and KT28 and their efficacy against *Pseudomonas aeruginosa* biofilm. *PLoS One* **10**: e0127603.

Danis-Wlodarczyk, K., Vandenheuvel, D., Jang, H. B., Briers, Y., Olszak, T., Arabski, M., Wasik, S., Drabik, M., Higgins, G., Tyrrell, J., Harvey, B. J., Noben, J. P., Lavigne, R., and Drulis-Kawa, Z. (2016) A proposed integrated approach for the preclinical evaluation of phage therapy in *Pseudomonas* infections. *Sci Rep* **6**: 28115.

Garbe, J., Bunk, B., Rohde, M. and Schobert, M. (2011) Sequencing and characterization of *Pseudomonas aeruginosa* phage JG004. *BMC Microbiol* **11**: a102.

Garbe, J., Wesche, A., Bunk, B., Kazmierczak, M., Selezska, K., Rohde, C., Sikorski, J., Rohde, M., Jahn, D. and Schobert, M. (2010) Characterization of JG024, a *Pseudomonas aeruginosa* PB1-like broad host range phage under simulated infection conditions. *BMC Microbiol* **10**: a301.

Heo, Y. J., Chung, I. Y., Choi, K. B., Lau, G. W. and Cho, Y. H. (2007) Genome sequence comparison and superinfection between two related *Pseudomonas aeruginosa* phages, D3112 and MP22. *Microbiology* **153**: 2885-2895.

Heo, Y. J., Lee, Y. R., Jung, H. H., Lee, J., Ko, G. and Cho, Y. H. (2009) Antibacterial efficacy of phages against *Pseudomonas aeruginosa* infections in mice and *Drosophila melanogaster*. *Antimicrob Agents Chemo* **53**: 2469-74.

Harvey, H., Bondy-Denomy, J., Marquis, H., Sztanko, K. M., Davidson, A. R. and Burrows, L. L. (2018) *Pseudomonas aeruginosa* defends against phages through type IV pilus glycosylation. *Nat Microbiol* **3**: 47-52.

Hertveldt, K., Lavigne, R., Pleteneva, E., Sernova, N., Kurochkina, L., Korchevskii, R., Robben, J., Mesyanzhinov, V., Krylov, V. N. and Volckaert, G. (2005) Genome comparison of *Pseudomonas aeruginosa* large phages. *J Mol Biol* **354**: 536-45.

Jarrell, K.F., Kropinski, A.M. (1981) Isolation and characterization of a bacteriophage specific for the lipopolysaccharide of rough derivatives of *Pseudomonas aeruginosa* strain PAO. *J Virol.* 38(2):529-538.

Koderi Valappil, S., Shetty, P., Deim, Z., Terhes, G., Urbán, E., Váczi, S., Patai, R., Polgár, T., Pertics, B. Z., Schneider, G., Kovács, T., and Rákhely, G. (2021) Survival Comes at a Cost: A coevolution of phage and its host leads to phage resistance and antibiotic sensitivity of *Pseudomonas aeruginosa* multidrug resistant strains. *Front Microbiol* **12**: 783722.

Kim, E. S., Bae, H. W. and Cho, Y. H. (2018a) A pilin region affecting host range of the *Pseudomonas aeruginosa* RNA phage, PP7. *Front Microbiol* **9**: a247.

Kim, S., Rahman, M., Seol, S. Y., Yoon, S. S., and Kim, J. (2012) *Pseudomonas aeruginosa* bacteriophage PA1Ø requires type IV pili for infection and shows broad bactericidal and biofilm removal activities. *Appl Environ Microbiol* **78**: 6380–6385.

Lavigne, R., Lecoutere, E., Wagemans, J., Cenens, W., Aertsen, A., Schoofs, L., Landuyt, B., Paeshuyse, J., Scheer, M., Schobert, M. and Ceyssens, P. J. (2013) A multifaceted study of *Pseudomonas aeruginosa* shutdown by virulent podovirus LUZ19. *mBio* **4**: e00061-13.

Le, S., He, X., Tan, Y., Huang, G., Zhang, L., Lux, R., Shi, W., and Hu, F. (2013) Mapping the tail fiber as the receptor binding protein responsible for differential host specificity of *Pseudomonas aeruginosa* bacteriophages PaP1 and JG004. *PloS One* **8**: e68562.

Li, L., Pan, X., Cui, X., Sun, Q., Yang, X. and Yang, H. (2016) Characterization of *Pseudomonas aeruginosa* phage K5 genome and identification of its receptor related genes. *J Basic Microbiol* **56**: 1344-1353.

Meadow, P. M. and Wells, P. L. (1978) Receptor sites for R-type pyocins and bacteriophage E79 in the core part of the lipopolysaccharide of *Pseudomonas aeruginosa* PAC1. *J Gen Microbiol* **108**: 339-343.

McCutcheon, J. G., Peters, D. L. and Dennis, J. J. (2018) Identification and characterization of type IV pili as the cellular receptor of broad host range *Stenotrophomonas maltophilia* bacteriophages DLP1 and DLP2. *Viruses* **10**: 338.

Olszak, T., Danis-Wlodarczyk, K., Arabski, M., Gula, G., Maciejewska, B., Wasik, S., Lood, C., Higgins, G., Harvey, B. J., Lavigne, R. and Drulis-Kawa, Z. (2019) PA5oct jumbo phage impacts planktonic and biofilm population and reduces its host virulence. *Viruses* **11**: 1089.

Ong, S. P., Azam, A. H., Sasahara, T., Miyanaga, K. and Tanji, Y. (2020) Characterization of *Pseudomonas* lytic phages and their application as a cocktail with antibiotics in controlling Pseudomonas aeruginosa. *J Biosci Bioeng* **129**: 693-699.

Patel, I. R. and Rao, K. K. (1983) Studies on the *Pseudomonas aeruginosa* PAO1 bacteriophage receptors. *Arch Microbiol* **135**: 155-157.

Pourcel, C., Midoux, C., Vergnaud, G. and Latino, L. (2017) A carrier state is established in *Pseudomonas aeruginosa* by phage LeviOr01, a newly isolated ssRNA levivirus. *J Gen Virol* **98**: 2181-2189.

Pourcel, C., Midoux, C., Vergnaud, G., and Latino, L. (2020) The basis for natural multi-resistance to phage in *Pseudomonas aeruginosa*. *Antibiotics* **9**: 339.

Pires, D. P., Dötsch, A., Anderson, E. M., Hao, Y., Khursigara, C. M., Lam, J. S., Sillankorva, S. and Azeredo, J. (2017) A genotypic analysis of five *P*. *aeruginosa* strains after biofilm infection by phages targeting different cell surface receptors. *Front Microbiol* **8**: a1229.

Pan, X., Cui, X., Zhang, F., He, Y., Li, L. and Yang, H. (2016) Genetic evidence for O-specific antigen as receptor of *Pseudomonas aeruginosa* phage K8 and its genomic analysis. *Front Microbiol* **7**: a252.

Rivera, M., Chivers, T. R., Lam, J. S. and McGroarty, E. J. (1992) Common antigen lipopolysaccharide from *Pseudomonas aeruginosa* AK1401 as a receptor for bacteriophage A7. *J Bacteriol* **174**: 2407-2411.

Roncero, C., Darzins, A. and Casadaban, M. J. (1990) *Pseudomonas aeruginosa* transposable bacteriophages D3112 and B3 require pili and surface growth for adsorption. *J Bacteriol* **172**: 1899-1904.

Shiley, J. R., Comfort, K. K. and Robinson, J. B. (2017) Immunogenicity and antimicrobial effectiveness of *Pseudomonas aeruginosa* specific bacteriophage in a human lung in vitro model. *Appl Microbiol Biotechnol* **101**: 7977-7985.

Temple, G. S., Ayling, P. D., and Wilkinson, S. G. (1986) Isolation and characterization of a lipopolysaccharide-specific bacteriophage of *Pseudomonas aeruginosa*. *Microbios* **45**: 81–91.

Vaca-Pacheco, S., Paniagua-Contreras, G. L., García-González, O. and de la Garza, M. (1999) The clinically isolated FIZ15 bacteriophage causes lysogenic conversion in *Pseudomonas aeruginosa* PAO1. *Curr Microbiol* **38**: 239-243.

Wright, R. C. T., Friman, V. P., Smith, M. C. M. and Brockhurst, M. A. (2019) Resistance evolution against phage combinations depends on the timing and order of exposure. *mBio* **10**: 01652-19.

Xuan, G., Lin, H., Tan, L., Zhao, G., and Wang, J. (2022) Quorum sensing promotes phage infection in *Pseudomonas aeruginosa* PAO1. *mBio* **13**: e0317421.

Yang, Y., Shen, W., Zhong, Q., Chen, Q., He, X., Baker, J. L., Xiong, K., Jin, X., Wang, J., Hu, F. and Le, S. (2020) Development of a bacteriophage cocktail to constrain the emergence of phage-resistant *Pseudomonas aeruginosa*. *Front Microbiol* **11**: a327.

Yokota, S., Hayashi, T. and Matsumoto, H. (1994) Identification of the lipopolysaccharide core region as the receptor site for a cytotoxin-converting phage, phi CTX, of *Pseudomonas aeruginosa*. *J Bacteriol* **176**: 5262-5269.

Zhang, F., Huang, K., Yang, X., Sun, L., You, J., Pan, X., Cui, X. and Yang, H. (2018) Characterization of a novel lytic podovirus O4 of *Pseudomonas aeruginosa*. *Arch Virol* **163**: 2377-2383.
